# The impact of human development on individual health: a causal mediation analysis examining pathways through education and body mass index

**DOI:** 10.7717/peerj.3053

**Published:** 2017-03-01

**Authors:** Aolin Wang, Onyebuchi A. Arah

**Affiliations:** 1 Department of Epidemiology, Fielding School of Public Health, University of California, Los Angeles, CA, United States; 2 California Center for Population Research (CCPR), Los Angeles, CA, United States; 3 UCLA Center for Health Policy Research, Los Angeles, CA, United States

**Keywords:** Human development, Body mass index, Mediation analysis, General health, Education, Pathways

## Abstract

**Background:**

The macro environment we live in projects what we can achieve and how we behave, and in turn, shapes our health in complex ways. Policymaking will benefit from insights into the mechanisms underlying how national socioeconomic context affects health. This study examined the impact of human development on individual health and the possible mediating roles of education and body mass index (BMI).

**Methods:**

We analyzed World Health Survey data on 109,448 participants aged 25 or older from 42 low- and middle-income countries with augmented human development index (HDI) in 1990. We used principal components method to create a health score based on measures from eight health state domains, used years of schooling as education indicator and calculated BMI from self-reported height and weight. We used causal mediation analysis technique with random intercepts to account for the multilevel structure.

**Results:**

Below a reference HDI level of 0.48, HDI was negatively associated with good health (total effect at HDI of 0.23: *b* =  − 3.44, 95% CI [−6.39–−0.49] for males and *b* =  − 5.16, 95% CI [−9.24,–−1.08] for females) but was positively associated with good health above this reference level (total effect at HDI of 0.75: *b* = 4.16, 95% CI [−0.33–8.66] for males and *b* = 6.62, 95% CI [0.85–12.38] for females). We found a small positive effect of HDI on health via education across reference HDI levels (*b* ranging from 0.24 to 0.29 for males and 0.40 to 0.49 for females) but not via pathways involving BMI only.

**Conclusion:**

Human development has a non-linear effect on individual health, but the impact appears to be mainly through pathways other than education and BMI.

## Introduction

“Wealthier nations are healthier nations” has long been known (Pritchett & Summers, 1996). The relationship between income and health, at both the individual and national levels, is perhaps one of the most documented findings in the population health literature (Preston, 1975; Pritchett & Summers, 1996; Hall, Barnes & Taylor, 2009). In low- and middle-income countries (LMICs), life expectancy has increased dramatically over the past century due to substantial achievements in control of infectious diseases via better sanitation and food safety, vaccines, antibiotics and improved nutrition (Schlipköter & Flahault, 2010). However, in the early 1990s, these countries started to experience rapid changes in lifestyle such as dietary and physical activity behaviors. Energy-dense poor quality diets and sedentary behaviors fueled an obesity epidemic (if not a pandemic), and an increased burden of nutrition-related non-communicable diseases (NCDs) was observed (Popkin, 1998; Hawkes, Chopra & Friel, 2009; Popkin, Adair & Ng, 2012). Though economic growth, increased individual income, and good health tend to go together, these nutritional and lifestyle changes and increased prevalence of NCDs may offset some of the health benefits of economic growth as countries go through economic and social development. Despite being studied using data from several decades ago (Preston, 1975; Pritchett & Summers, 1996), the effect of (macro socioeconomic) development on health will benefit from re-examination in the LMICs using health measures other than mortality and life expectancy, and with a view to explore potential mechanisms such as mediation and interaction.

The underlying mechanisms are important for understanding the overall effect of development on health. Many studies on how education affects our health exist (Zimmerman, Woolf & Haley, 2015). Investing in schooling, particularly for girls, is one of the key strategies for developing countries to promote health and economic growth (World Bank, 1993) and could be an important intermediate factor on the pathway. Education also interacts with a country’s level of human development (Van der Kooi et al., 2013) and country context (Rehkopf, Dow & Rosero-Bixby, 2010) in affecting health. Adoption of health behaviors is another potential mediator in the mechanism connecting country development and health. Obesity, a crucial indicator closely related to health behavior, has been linked to various adverse health outcomes (James et al., 2004; Wang, Stronks & Arah, 2014; Wang & Arah, 2015). Moreover, its consequences appear to be nuanced for people with different educational attainments and from countries at various stages of development (Wang, Stronks & Arah, 2014).

Indeed, we cannot discuss a person’s health without considering the ‘contextual web’ the person is in Arah (2009). The macro environment we live in projects what we can achieve and how we behave, and in turn, shapes our health in a complex way. Interesting insights can be gained from examining the extent to which the macro environment shapes health via mechanisms involving education, obesity, and other intermediate factors. This study aimed at addressing these mediation (mechanism) questions. More specifically, we used global data to investigate (i) the impact of national human development level as measured by human development index on individual health, and (ii) the mediating roles of both education and body mass index in the relation between human development and health.

## Methods

### Study sample and variables

We used the World Health Survey (WHS) data from 49 low- and middle-income countries. Conducted by the WHO from 2002 to 2004, the WHS used a standardized methodology that provided a basis for examining individual health measures across countries (WHO, 2013). Within each country, samples were probabilistically selected with every individual being assigned to a known non-zero selection probability. These samples were nationally representative except in China, Comoros, Congo, Côte d’Ivoire, India, and the Russian Federation, where the survey was carried out in geographically limited regions. This study included participants from fourteen countries in the African region, nine in the European region, seven in the Americas, five in the South-East Asia region, five in the Western Pacific region, and two in the Eastern Mediterranean region (Table S2). All respondents were interviewed face-to-face with the standardized WHS survey, which included questions regarding demographic, socioeconomic, and behavioral factors (WHO, 2013; Moussavi et al., 2007).

#### Outcome

Individuals were asked to report their perceived difficulties based on a 5-point Likert scale question for eight health state domain (two questions per domain): mobility, self-care, pain and discomfort, cognition, interpersonal activities, vision, sleep and energy, and affect (WHO, 2013). The health state measures have been extensively tested (Üstün et al.) and have shown good consistency and reliability (Moussavi et al., 2007). We performed a factor analysis using polychoric correlations to account for the covariance structure of the responses to individual questions. Similar to a previous study (Moussavi et al., 2007), we chose the one-factor solution based on the large eigenvalue of the first factor (8.85, 73% as a cumulative percentage of the variance explained) and the high communalities of the original variables (between 0.36 and 0.69). Then, we used the principal components method for factor extraction and the regression scoring method to obtain the factor scores. The factor score was rescaled to go from 0 (indicating worst health) to 100 (indicating best health).

#### Exposure

We chose the Human Development Index (HDI) as a measure of the national socioeconomic environment for human development in a country. HDI is a unit-free index between zero and one that is calculated based on life expectancy at birth, adult literacy rate, combined gross enrollment ratio for primary, secondary and tertiary education, and GDP per capita for each country. In this study, we used HDI reported for 1990 (United Nations Development Programme, 2013) and rescaled the score to range from zero to ten. We lagged the HDI for more than ten years to capture its effect on shaping individual education and to minimize reverse causation. We also assumed that HDI from 1990 was a good indicator of the national socioeconomic environment for the period leading up to 1990.

#### Mediators

Individual education was measured by the years of schooling (including higher education).

Body mass index (BMI) was defined as an individual’s self-reported weight (kg) divided by self-reported height squared (m^2^). We excluded participants with height less than 1.22 m (*n* = 1, 174) or greater than 2.11 m (*n* = 27), and participants with a weight that was 3 SDs above (*n* = 821) or 2 SDs below (*n* = 382) the crude sample mean of 63.6 kg. We further excluded individuals with BMI less than 14 kg/m^2^ (*n* = 252).

#### Confounders

Potential confounders we included were WHO region, individual age, and sex. In sensitivity analyses, we further considered potential confounders of the BMI-health relationship that were possibly influenced by education or HDI: living in urban areas, unemployment, marital status, and health behaviors such as smoking, alcohol use, and physical activity.

### Conceptual framework

**Figure 1:**
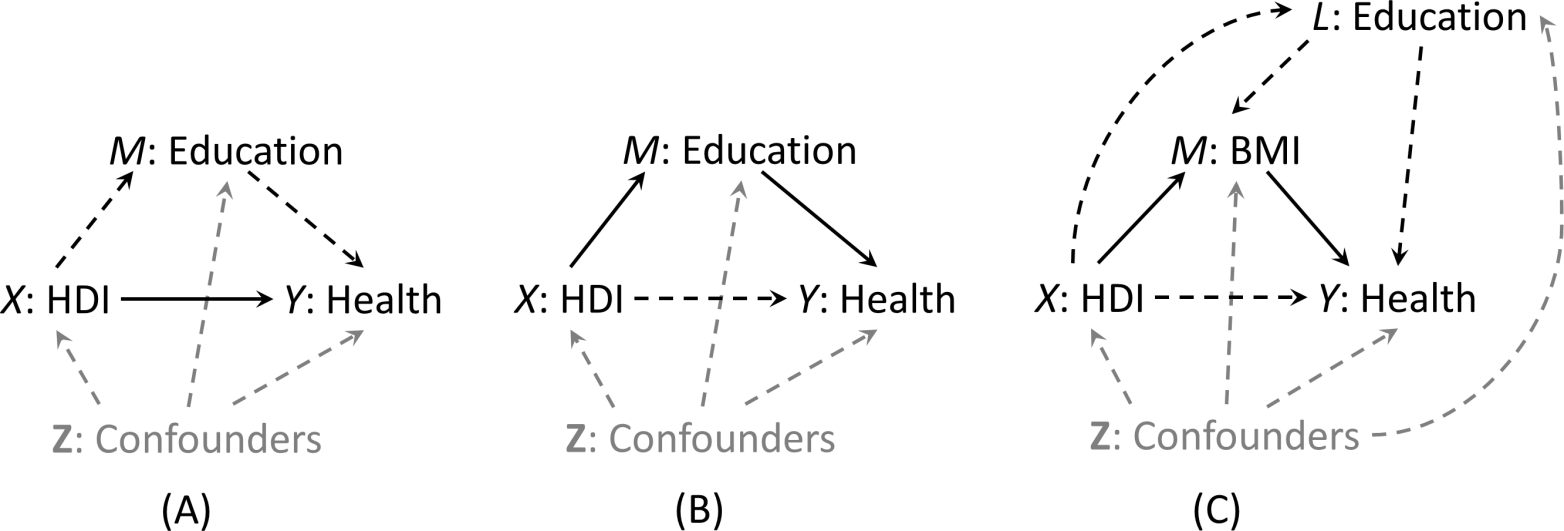
Graphical representation (solid lines) of pure direct effect of HDI on health (A) and total indirect effect of HDI via education (B) when education is the mediator of interest (Scenario 1), and natural indirect effect of HDI via BMI only (C) when BMI is the mediator of interest (Scenario 2). In scenario 1, the BMI path-specific effect was incorporated in the pure direct effect.

**Figure 2:**
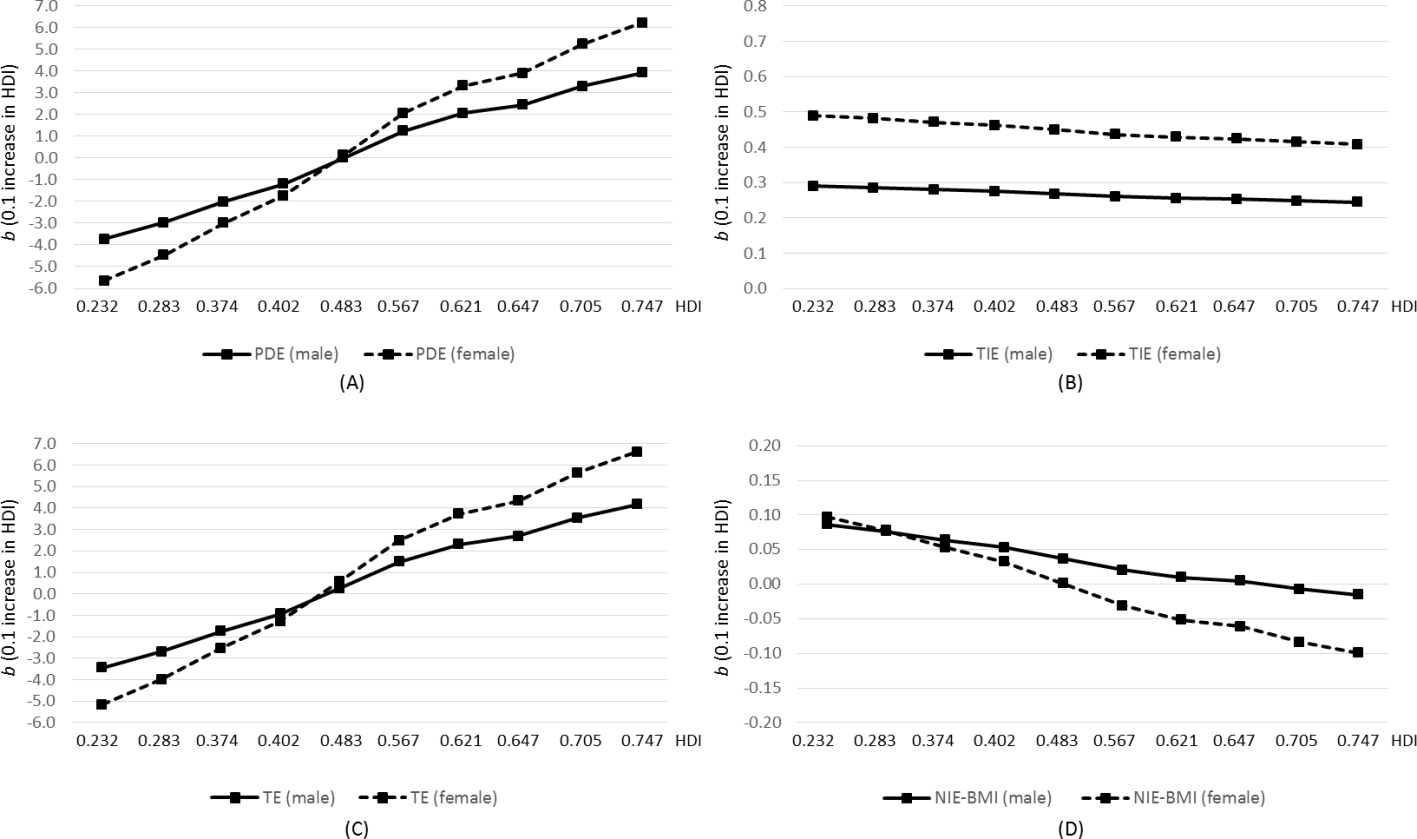
Pure direct effect (PDE; A), total indirect effect (TIE; B), and total effect (TE; C) of HDI on health when education is the mediator of interest in Scenario 1, and natural indirect effect via BMI only in Scenario 2 (D), obtained from multilevel regression analysis of the World Health Survey 2002–2004 (*N* = 109,448). *Y* axis represents mean difference in health score associated with a 0.1-unit increase in HDI. *X* axis represents selected reference HDI values within the range of the current sample.

We aimed to examine the path-specific effects under the following two scenarios where education (scenario 1) or BMI (scenario 2) was the mediator of interest, respectively. In scenario 1, the pure direct effect captured the impact of human development on individual health through pathways other than individual-level education (Fig. 1A) whereas the total indirect effect measured such impact through education (Fig. 1B). In examining the mediating role of BMI, a consequence of individual-level education, we further decomposed the pure direct effect of HDI on health into (1) the HDI effect through BMI but not education (i.e., the BMI-path-specific effect as presented in Fig. 1C), and (2) the natural direct effect of HDI on health through neither education nor BMI. Detailed assumptions used for effect identification can be found in Supplemental Information 1.

### Statistical analysis

We used appropriate descriptive statistics to summarize the characteristics of the participants by sex and WHO region. Under the assumptions of general consistency, conditional exchangeability (no-uncontrolled-confounding), and positivity (Vanderweele, Vansteelandt & Robins, 2014), we can estimate different types of effects via regression models. Effect definitions and their empirical expressions are listed in Table S1. We included an HDI squared term into the outcome regression model to capture the non-linearity of the effect of HDI on individual health. To account for the within-country clustering, we used PROC NLMIXED procedure to fit multilevel generalized linear models with country-specific random intercept for each model of health score, BMI, and education. The NLMIXED procedure fits nonlinear mixed models by maximizing an approximation to the likelihood integrated over the random effects, using adaptive Gaussian quadrature (default). Users have the flexibility to specify a conditional distribution for the data (given the random effects) to have either a standard form (normal, binomial, Poisson) or a general distribution that you code using SAS programming statements (SAS Institute Inc., 2008). We reported effect estimates and the corresponding 95% confidence intervals (95% CIs) for males and females separately. All analyses were conducted in SAS 9.4 (SAS Institute Inc., Cary, North Carolina, USA).

### Sensitivity analysis

For scenario 1, we relaxed the sample restriction criteria to include individuals with complete information on HDI, education, and health score (*N* = 148, 679) and re-estimated the pure direct effect, total indirect effect and total effect. For scenario 2, we further examined the robustness of our results to the presence of intermediate confounders ***V*** (affected by HDI, education or both) of the BMI-health relationship including living in urban areas, marital status, unemployment, smoking, alcohol use, and physical inactivity. We used a g-estimation-like (or a substitution) method to create a confounding-free outcome variable where this new outcome variable was independent of ***V*** conditional on HDI, education, BMI, and other covariates (the set ***Z***). Details can be found in the Supplemental Informations 1 and 2.

## Results

Among 195,808 participants aged 25 years or older, 109,448 (55.9%) participants from 42 countries had complete information on all covariates. Country-specific sample size and characteristics are presented in the (Table S2). We excluded participants from Burkina Faso, Chad, Comoros, Ethiopia, Herzegovina, and Georgia (*N* = 15,770) because of missing country’s HDI measures.

Table 1 shows participant characteristics by sex and WHO region. Human development index varied by WHO region, with the European region having the highest mean HDI (0.71) and the South-East Asia region the lowest (0.44). Participants from the Europe region were oldest (mean age: 48.0 for males and 49.0 for females) and most educated (mean years of schooling: 12.4 for males and 12.2 for females), and had the highest mean BMI values (25.7 for males and 25.9 for females) but the lowest health score (86.4 for males and 82.2 for females). Except for the European region and the region of the Americas, participants from other WHO regions had similar sex-specific mean age, education years, BMI, and health scores. Overall, females were less educated and reported poorer health. A descriptive table (Table S3) on participants with complete information on HDI, education, and health score only can be found in Supplemental Information 1; it revealed similar patterns.

**Table 1:** Participant characteristics by WHO region, World Health Survey 2002–2004 (*N* = 109, 448).

Characteristics, mean (SD)	Africa	The Americas	Eastern Mediterranean	Europe	South-East Asia	Western Pacific	All
**Male**							
Total, *N* (%)	9,873 (19.6)	16,072 (31.8)	2,710 (5.4)	4,562 (9)	8,020 (15.9)	9,273 (18.4)	50,510 (100)
Human development index	0.47 (0.11)	0.63 (0.05)	0.49 (0.08)	0.71 (0.02)	0.44 (0.10)	0.54 (0.09)	0.55 (0.12)
Age, years	42.2 (14.1)	45.8 (15.3)	42.9 (13.6)	48.0 (15.1)	43.3 (13.4)	43.6 (13.2)	44.3 (14.4)
Education, years	7.5 (5.2)	7.1 (5.1)	7.3 (5.9)	12.4 (3.4)	6.9 (4.8)	7.8 (4.3)	7.8 (5.1)
Body mass index, kg/m^2^^a^	23.4 (4.0)	25.4 (3.8)	23.9 (3.9)	25.7 (3.3)	21.2 (3.3)	22.4 (3.4)	23.7 (4.0)
Health score	88.2 (14.2)	90.9 (11.3)	91.3 (12.6)	86.4 (13.2)	87.9 (14.1)	88.7 (13.5)	89.1 (13.1)
**Female**							
Total, *N* (%)	11,327 (19.2)	19,789 (33.6)	2,051 (3.5)	8,373 (14.2)	7,121 (12.1)	10,277 (17.4)	58,938 (100)
Human development index	0.46 (0.1)	0.63 (0.05)	0.51 (0.08)	0.71 (0.02)	0.44 (0.11)	0.54 (0.09)	0.56 (0.12)
Age, years	41.9 (14.5)	45.0 (15.2)	42.2 (13.8)	49.0 (15.3)	43.0 (13.6)	42.9 (13.3)	44.3 (14.7)
Education, years	5.7 (5.0)	6.9 (5.1)	4.8 (5.7)	12.2 (3.5)	5.3 (4.8)	7.1 (4.6)	7.2 (5.2)
Body mass index, kg/m^2^^a^	24.1 (4.9)	25.8 (4.7)	24.6 (4.5)	25.9 (4.5)	21.2 (3.7)	22.1 (3.9)	24.2 (4.8)
Health score	84.2 (15.7)	87.5 (12.7)	86.9 (15.4)	82.2 (14.9)	85.2 (15.8)	87.4 (13.9)	85.8 (14.5)

**Notes.**

a Statistics for body mass index were from the sample restricted to individuals who had normal height and weight status and those whose BMI < 14 kg/m^2^ (*N* = 109,448).

Sex-specific mean differences in health score associated with a 0.1-unit increase in HDI at the median HDI level (comparing HDI = 0.672 to HDI = 0.572) or at multiple reference HDI levels are presented in Table 2 and Fig. 2 respectively. At the median HDI level of 0.572 in scenario 1, increase in HDI was positively associated with better health in both males (*b* = 1.58, 95% CI [−0.61–3.77]) and females (*b* = 2.61, 95% CI [−0.09–5.32]). The impact appeared to be mostly through pathways other than individual-level education (male: *b* = 1.32, 95% CI [−0.87–3.51]; female: *b* = 2.18, 95% CI [−0.52–4.88]). A small positive indirect effect of HDI via education was seen in both males (*b* = 0.26, 95% CI [0.17–0.35]) and females (*b* = 0.44, 95% CI [0.28–0.59]). The BMI-path-specific effect of HDI was almost null in both sexes (male: *b* = 0.016, 95% CI [−0.005–0.037]; female: *b* =  − 0.033, 95% CI [−0.077–0.011]). All types of effects of HDI on health depended on the reference value of HDI. An increase in HDI below a reference HDI level of 0.483 was negatively associated with good health (total effect at HDI of 0.232: *b* =  − 3.44, 95% CI [−6.39–−0.49] for males and *b* =  − 5.16, 95% CI [−9.24–−1.08] for females) but was positively associated with good health above this reference level (total effect at HDI of 0.747: *b* = 4.16, 95% CI [−0.33–8.66] for males and *b* = 6.62, 95% CI [0.85–12.38] for females). This pattern for the total effect was also seen in pure direct effect. We found a small positive effect of HDI on health via education across reference HDI levels (*b* ranging from 0.24 to 0.29 for males and 0.40 to 0.49 for females) but not via pathways involving BMI only. As HDI increased, both mediated effects via education or via BMI only decreased slightly. The effect size among females was larger than that among males. Since the effect of HDI on health can be modeled by a quadratic regression model, in the (Fig. S1) we present an alternative graph depicting the HDI effect when comparing countries with different HDI to Ghana or China (with reference HDI of 0.502). The further the HDI value was from the reference HDI of 0.502, the larger the effect size was. Accordingly, the total effect and the pure direct effect of HDI on health were U-shaped when comparing HDI of different countries to the reference HDI value (of Ghana or China).

**Table 2:** Effect estimate (95% Confidence Interval) for human development level (comparing 0.672–0.572) on individual health, World Health Survey 2002–2004 (*N* = 109,448).

	Male *b* (95% CI)	Female *b* (95% CI)
**Scenario 1**^a^		
Total effect	1.58 (−0.61, 3.77)	2.61 (−0.09, 5.32)
Pure direct effect	1.32 (−0.87, 3.51)	2.18 (−0.52, 4.88)
Total indirect effect	0.26 (0.17, 0.35)	0.44 (0.28, 0.59)
**Scenario 2**^b^		
Natural indirect effect via BMI only	0.016 (−0.005, 0.037)	−0.033 (−0.077, 0.011)

**Notes.**

a Education is the mediator of interest.

b BMI path-specific effect, part of the pure direct effect in Scenario 1, was further examined.

Sensitivity analyses using a less restricted sample revealed similar point estimates but narrower confidence intervals (Table S4 and Fig. S2). Summary statistics for potential intermediate confounders are presented in the (Table S5). After accounting for the intermediate confounders (Fig. S3), effect estimates for the natural indirect effect via BMI only (Fig. S4) were similar to those from the main analyses.

## Discussion

This study examined the impact of national human development on individual health and possible pathways via education and BMI, using large population data. We found that the HDI effect on health depended on the reference HDI level: the total effect and pure direct effect of HDI were negative at low HDI level but became positive at the higher level of HDI. The HDI effect on individual-level health was mainly through pathways other than education and BMI. The impact of HDI on health was greater for females than for males.

Our study found that the effect of country-level human development on individual health was non-linear. This indicated that at the lower end of the HDI spectrum, people in countries with higher human development level tended to have poorer health whereas, at the upper end of this spectrum, higher human development level predicted better individual health. Around the median level of HDI, people from countries that were 0.1-unit apart in HDI tended to have similar health status. The HDI assessed how well countries are doing in three dimensions: health, education, and living standards (United Nations Development Programme, 2013). The overall health status and educational achievement for residents in a country will tend to grow hand in hand with the country’s adult literacy rate and combined gross enrollment ratio for primary, secondary and tertiary education. Yet improved living standards, as captured by gross national income per capita, may have affected people’s health in a complex way: the net health benefit may depend on the interrelationships of various factors including but not limited to improved social infrastructure, public health interventions, technology, and lifestyle changes due to urbanization and globalization. Low- and middle-income countries continue to experience epidemiological transitions from infectious diseases to chronic NCDs. NCDs continue to play increasingly important roles in personal health, especially for older people. In addition, the fast economic growth in some countries may be at the expense of a positive health-supporting environment. All these aspects contributed to the complicated relationship between development and health.

Our result may reflect investments in and prioritization of different aspects in overall health improvement as these countries went through various stages of economic and social development. Such investment may touch all aspects of health and health care: building roads for improving access to care, providing effective treatment for HIV/AIDS, tuberculosis, and malaria, promoting vaccination and proper use of antibiotics, strengthening primary care and preventive interventions and so on. Though all these investments and efforts may have greatly increased life expectancy at birth at the country level, they may differentially correlate with the self-reported health measures that were used in the current study and were heavily dependent on the presence of chronic disease. There can be lessons that we can learn from countries at the lower end of the human development spectrum. People from these countries achieved similar or even higher health status compared to people from countries with median health development level, possibly attributable to their targeted health investments and not being affected by the nutrition transition when the study was conducted.

Past work has documented the presence of effect modification of the relationship between education and health by HDI (Van der Kooi et al., 2013), and of the relationship between obesity and health outcomes by education (Schafer & Ferraro, 2011; Wang, Stronks & Arah, 2014). Incorporating such interactions is crucial for the present study. Despite the relatively small effect size here, we found a consistent positive indirect effect of HDI on health through increased education at each reference HDI level. In countries with lower human development levels, the education channel offset part of the negative impact of increased development on health. In countries with higher human development levels, education contributed to the overall positive impact of development on health. However, the pathways through BMI but not education did not appear to play an important role in transmitting the impact of development on health. Possible explanations included that the impact of development via BMI also went through education and other upstream variables, or that the mediating role of BMI depended on HDI and education in a complex way that the current model cannot capture. Future studies could explore pathways through other factors such as neighborhood environment, health care quality, and access to care (Adler & Stewart, 2010).

Though women suffer more from ill health than men do (Idler, 2003; Moussavi et al., 2007), our study suggests that women could potentially have more gains in health than men could as a country achieves higher human development. At a low human development level, national human development had a more negative impact for women than for men. The underlying mechanisms for the sex difference are still unclear. Women have been found to be at a higher risk of depression (Patel, 2001). In the current study sample, depression status is highly predictive of poor health (Moussavi et al., 2007). It may be that the level of human development correlates with mental health services and women from countries with good mental health care (usually countries with higher human development) gain more in terms of health compared to their male counterparts.

The standardized methodology used in the WHS allowed for pooled analyses from middle- and low-income countries across continents. The use of causal mediation analysis enabled us to incorporate nonlinear relationships such as previously documented interactions between HDI and education (Van der Kooi et al., 2013), and between education and BMI (Wang, Stronks & Arah, 2014) in affecting individual health outcomes. We conducted sensitivity analysis to check the robustness of the BMI-path-specific effect against the presence of confounders of the BMI-health relationship affected by HDI or education. We used linear mixed models to account for the multilevel structure of the data and adjusted for both contextual and individual confounders.

Several methodological limitations need to be addressed. We imposed temporality assumptions on the cross-sectional WHS data: individual-level education preceded BMI measurement, which preceded health status at the time of the survey. This is a reasonable assumption since formal education usually happens before age 25. However, there is still a slight chance that middle-age health status affected the cumulative education years. BMI value tends to be stable at middle age, but we cannot rule out the possibility of reverse causation for BMI-health relationship. Despite sensitivity analyses against intermediate confounding, our result for the BMI-path-specific effect of HDI could still be subject to uncontrolled confounding between BMI and health. There could be measurement error in BMI that was created based on self-report height and weight.

## Conclusions

Our study provides an update on the effect of development on health and further examined the underlying pathways through education and BMI. The impact of development on health depended on the level of national development. The effect of national human development on individual health was mainly through pathways other than education and BMI, and it differed by sex. Country-level development may harm or benefit human health, which has future implications for human development (Bloom & Canning, 2003; Jamison, 2006). Characterizing the impact of human development on health can help shed light on how policymakers could translate economic growth into health for all.

##  Supplemental Information

10.7717/peerj.3053/supp-1 Supplemental Information 1 Supplemental file

10.7717/peerj.3053/supp-2 Supplemental Information 2 Analysis program

## Additional Information and Declarations

### Data Availability

The following information was supplied regarding data availability: The World Health Survey data are publicly available at http://apps.who.int/healthinfo/systems/surveydata/index.php/catalog (registration required). The SAS program for the main analyses is available at: https://figshare.com/s/8a03ec6afc06ae81b6cb.
