# Supplementary material for: The impact of human development on individual health: a causal mediation analysis examining pathways through education and body mass index
Source: PeerJ. 2017 Mar 1;5:e3053. doi: 10.7717/peerj.3053 (PMC5335675; doi:10.7717/peerj.3053)
Supplement: Supplemental Information 1 [file peerj-05-3053-s001.pdf]

Supplementary Materials

Title:

The impact of human development on individual health: a causal mediation analysis examining pathways through education and body mass index

Aolin Wang[1,2*] and Onyebuchi A. Arah[1-3]

[1]Department of Epidemiology, Fielding School of Public Health, University of California, Los Angeles (UCLA), Los Angeles, California, USA

[2]California Center for Population Research (CCPR), Los Angeles, California, USA

[3]UCLA Center for Health Policy Research, Los Angeles, California, USA

[*]Corresponding author: Aolin Wang

Email: aolinw@ucla.edu

Address: Department of Epidemiology, UCLA Fielding School of Public Health, 650 Charles E. Young Drive South, Los Angeles, CA 90095-1772, USA.

1. Assumptions for identification

Notation

Let *Y* denote one's health state, *X* the country's human development level, *M* the mediator of interest: education in scenario 1 and BMI in scenario 2, *L* the exposure-induced mediator-outcome confounder: education in scenario 2. Let $Y(x, M(x))$ denote the potential *Y* had *X* been set to *x*, *M* been set to the natural value under $X=x$ and $Y(x, L(x), M(x, L(x)))$ denote the potential *Y* had *X* been set to *x*, *L* been set to the natural value under $X=x$, *M* been set to the natural value under $X=x$ and $L = L(x)$. Let x_1 (index) and x_0 (reference) represent two exposure values we wish to compare.

To estimate the effects defined in Table 1 using observational data, we assumed stable unit treatment value assumptions (SUTVA) (Rubin, 1980, 1990), general consistency, conditional exchangeability (no-uncontrolled-confounding), and positivity (Daniel et al., 2015). The conditional exchangeability assumption for natural effects included: (i) no uncontrolled confounding of the $(X, M) – Y$ and $X – M$ relationship given covariate set $\boldsymbol{Z}$, and (ii) no members of the covariate set $\boldsymbol{Z}$ are affected by *X* in scenario 1. In scenario 2, we assumed that (iii) no uncontrolled confounding of the $(X, L, M) – Y$, $(X, L) – M$, and $L – M$ relationship given covariate set $\boldsymbol{Z}$, and (iv) no members of the covariate set $\boldsymbol{Z}$ are affected by *X or L.* Violation of assumption (ii) in scenario 2 – confounders for the *M–Y* relationship being affected by either X or L or both – will be examined in sensitivity analysis.

2. G-estimation-like method for sensitivity analysis

We examined how the BMI path-specific effect in scenario 2 will change under possible violation of assumption (iv) above (Figure S2). We hypothesized that living in urban or rural areas, unemployment, marital status, smoking, alcohol use, and physical inactivity could be confounders of the BMI – health relationship. These factors were influenced by HDI, education, or both and were denoted using $\boldsymbol{V}$. In a structure presented in Figure S2 (A), not adjusting for $\boldsymbol{V}$ will result in bias for the BMI-path-specific effect of HDI because of the extra path HDI→intermediate confounders→health. However, we cannot use traditional regression adjustment because adjusting for $\boldsymbol{V}$ will block the path HDI→intermediate confounders→BMI→health, which is part of the indirect effect of HDI via BMI but not education. In this case, using this g-estimation-like technique (or a substitution estimator) to de-activate the path from intermediate confounders to health will be appropriate for unbiased estimation of this targeted effect as presented in Figure S2 (B). We thus created the new outcome variable Y^* where $Y^* \perp \boldsymbol{V} \mid X, L, M, \boldsymbol{Z}$:

$Y^* = Y - \varphi_V \cdot \boldsymbol{v} + \varphi_V \cdot E(\boldsymbol{V})$ where φ_V came from the model for Y with additional adjustment for $\boldsymbol{V}$

$E(Y|x, m, \boldsymbol{z}, \boldsymbol{v}; \varphi) = \varphi_Y + \varphi_X \cdot x + \varphi_{X^2} \cdot x^2 + \varphi_L \cdot l + \varphi_M \cdot m + \varphi_{XL} \cdot x \cdot l + \varphi_{XM} \cdot x \cdot m + \varphi_{LM} \cdot l \cdot m + \varphi_Z \cdot \boldsymbol{z} + \varphi_V \cdot \boldsymbol{v}$ and $E(\boldsymbol{V})$ represented the crude expected value for $\boldsymbol{V}$. Then, we used this new Y^* variable for all analyses.

Supplementary Tables

Table S1 Effect (counterfactual) definitions and empirical expressions, applied to the World Health Survey 2002-2004 data.

Effect	Counterfactual definition[a]	Empirical expression[b]
**Scenario 1**	X: HDI, M: education years, Y: health<br>$E(M \mid x, z^M; \delta) = \delta_M + \delta_X \cdot x + \delta_Z \cdot z^M$<br>$E(Y \mid x, m, z; \theta) = \theta_Y + \theta_X \cdot x + \theta_{X^2} \cdot x^2 + \theta_M \cdot m + \theta_{XM} \cdot x \cdot m + \theta_Z \cdot z$	
TE[c]	E[$Y(x_1, M(x_1))$<br>$- Y(x_0, M(x_0))$]	$[\theta_X + \theta_M \cdot \delta_X + \theta_{XM} \cdot (\delta_M + \delta_X \cdot (x_1 - x_0) + \delta_Z \cdot z^M)] \cdot (x_1 - x_0) + \theta_{X^2} \cdot (x_1{}^2 - x_0{}^2)$
PDE	E[$Y(x_1, M(x_0))$<br>$- Y(x_0, M(x_0))$]	$[\theta_X + \theta_{XM} \cdot (\delta_M + \delta_X \cdot x_0 + \delta_Z \cdot z^M)] \cdot (x_1 - x_0) + \theta_{X^2} \cdot (x_1{}^2 - x_0{}^2)$
TIE	E[$Y(x_1, M(x_1))$<br>$- Y(x_1, M(x_0))$]	$[\theta_M \cdot \delta_X + \theta_{XM} \cdot \delta_X \cdot x_1] \cdot (x_1 - x_0)$
**Scenario 2**	X: HDI, L: education years, M: BMI, Y: health<br>$E(L \mid x, z^L; \alpha) = \alpha_L + \alpha_X \cdot x + \alpha_Z \cdot z^L$<br>$E(M \mid x, z^M; \beta) = \beta_M + \beta_X \cdot x + \beta_L \cdot l + \beta_Z \cdot z^M$<br>$E(Y \mid x, m, z; \gamma) = \gamma_Y + \gamma_X \cdot x + \gamma_{X^2} \cdot x^2 + \gamma_L \cdot l + \gamma_M \cdot m + \gamma_{XL} \cdot x \cdot l + \gamma_{XM} \cdot x \cdot m + \gamma_{LM} \cdot l \cdot m + \gamma_Z \cdot z$	
$\text{NIE}_{X \to M \to Y}$	E[$Y(x_1, L(x_0), M(x_1, L(x_0)))$ –<br>$Y(x_1, L(x_0), M(x_0, L(x_0)))$]	$\beta_X \cdot [\gamma_M + \gamma_{LM} \cdot \alpha_L + \gamma_{XM} \cdot x_1 + \gamma_{LM} \cdot \alpha_X \cdot x_0 + \gamma_{LM} \cdot \alpha_Z \cdot z^L] \cdot (x_1 - x_0)$

[a] We used x_1 (index) and x_0 (reference) to denote the two exposure values that we wish to compare.

[b] The average effect were conditional on covariates Z=z. We used z^M, z^L and z to denote the set of covariates included in the model for M, L, and Y respectively.

[c] TE: total effect of HDI on health, PDE: pure direct effect, TIE: total indirect effect of HDI on health via education, $\text{NIE}_{X \to M \to Y}$: natural indirect effect of HDI on health via BMI but not education.

Table S2 Country-specific sample size, percent female, mean age, and country's human development index; World Health Survey 2002-2004.

Country	Initial sample size	Human development index 1990	N missing health score	N missing education	Final N (appendix result, scenario 1)	N missing height or weight	Final N (main result)	Female (%)	Mean age
**African Region (AFR)**									
Burkina Faso	3605	Missing	91	749	0	2328	0	50.7	41.4
Chad	3624	Missing	364	1177	0	748	0	51.7	41.8
Comoros	1411	Missing	55	30	0	14	0	57.0	47.5
Congo	1935	0.553	413	1170	673	225	651	52.4	40.0
Côte d'Ivoire	2398	0.380	248	928	1353	222	1255	42.0	40.3
Ethiopia	3772	Missing	442	2381	0	3138	0	51.1	41.9
Ghana	3292	0.502	113	339	2855	214	2609	55.6	45.1
Kenya	3441	0.471	46	256	3144	295	2842	57.9	42.6
Malawi	3690	0.283	124	371	3203	213	2956	56.5	42.3
Mali	3095	0.232	2389	2623	90	1056	35	43.2	46.2
Mauritania	3008	0.367	294	854	1942	437	1558	61.7	43.2
Mauritius	3385	0.621	302	42	3045	1239	1867	52.7	45.2
Namibia	3283	0.577	1200	352	1947	321	1782	59.3	42.6
Senegal	2527	0.384	548	1381	956	1110	590	48.2	42.9
South Africa	1869	0.619	330	0	1539	624	866	53.2	41.8
Swaziland	2364	0.538	851	748	1307	940	1002	53.8	43.8
Zambia	2839	0.407	333	87	2424	1062	1502	53.5	41.2
Zimbabwe	3013	0.488	117	48	2863	1014	1685	64.9	43.1
**Region of the Americas(AMR)**									
Brazil	4209	0.612	548	42	3622	480	3194	56.8	45.6
Dominican Republic	3758	0.589	61	17	3685	1199	2458	53.2	45.8
Ecuador	3866	0.643	326	461	3126	444	2648	55.7	45.0
Guatemala	3822	0.483	143	754	2955	1140	2096	61.1	44.6
Mexico	32129	0.647	0	0	32129	12689	19272	57.5	45.1
Paraguay	4062	0.581	45	1	4017	357	3597	54.6	44.9
Uruguay	2680	0.691	22	5	2654	2	2596	51.8	48.7
**Eastern Mediterranean Region (EMR)**									
Morocco	4184	0.459	4184	2473	0	2538	0	58.3	44.9
Pakistan	5027	0.402	190	696	4178	2400	1957	45.3	41.6
Tunisia	4213	0.567	344	527	3411	737	2804	54.9	45.9
**European Region (EUR)**									
Bosnia and Herzegovina	917	Missing	386	1	0	3	0	58.3	50.1
Croatia	932	0.689	20	4	909	8	885	59.9	54.1

Table S2 Country-specific sample size, percent female, mean age, and country's human development index; World Health Survey 2002-2004.

**Country**	**Initial sample size**	**Human development index 1990**	**N missing health score**	**N missing education**	**Final N (appendix result, scenario 1)**	**N missing height or weight**	**Final N (main result)**	**Female (%)**	**Mean age**
Czech Republic	828	0.762	90	16	724	18	688	55.6	51.3
Estonia	927	0.73	41	3	884	5	849	63.8	52.3
Georgia	2441	Missing	16	8	0	7	0	57.9	52.2
Hungary	1262	0.701	315	0	947	17	906	59.4	53.0
Kazakhstan	4110	0.686	104	9	3997	330	3621	65.8	43.3
Latvia	763	0.71	72	18	679	116	568	68.3	54.6
Russian Federation	4068	0.729	278	174	3629	869	2838	64.6	54.0
Slovakia	1917	0.747	680	585	1222	585	1185	63.8	43.8
Ukraine	2517	0.705	205	37	2275	920	1395	65.3	50.8
**South-East Asia Region (SEAR)**									
Bangladesh	4526	0.382	821	1380	2633	3891	500	52.2	42.6
India	8139	0.431	1640	1371	5492	1911	4196	51.7	43.0
Myanmar	4996	0.347	4	0	4992	30	4946	57.3	44.6
Nepal	6979	0.388	49	426	6511	4694	2146	56.3	43.3
Sri Lanka	5642	0.62	710	673	4409	1328	3353	54.0	44.9
**Western Pacific Region (WPR)**									
China	3674	0.502	54	17	3603	4	3579	51.4	47.2
Lao People's Democratic Republic	4060	0.395	86	5	3969	9	3923	52.7	41.8
Malaysia	5249	0.641	203	153	4909	999	3910	56.8	44.2
Philippines	8378	0.591	110	24	8245	1582	6614	54.6	42.6
Viet Nam	2982	0.476	1427	35	1532	7	1524	55.5	43.4

Table S3 Participant characteristics by WHO region, World Health Survey 2002-2004 (N=148679).

**Characteristics**, mean (SD)	**Africa**	**The Americas**	**Eastern Mediterranean**	**Europe**	**South-East Asia**	**Western Pacific**	**All**
**Male**							
Total, N (%)	12216 (18.6)	22724 (34.6)	3926 (6.0)	5419 (8.2)	11250 (17.1)	10201 (15.5)	65736 (100)
Human development index	0.47 (0.11)	0.63 (0.04)	0.47 (0.08)	0.71 (0.02)	0.43 (0.09)	0.54 (0.09)	0.55 (0.12)
Age, years	42.7 (14.4)	45.7 (15.4)	43.4 (14.2)	48.7 (15.3)	44.3 (14)	43.9 (13.4)	44.7 (14.7)
Education, years	7.3 (5.1)	7.1 (5.1)	6.6 (5.9)	12.3 (3.5)	6.0 (4.9)	7.7 (4.3)	7.4 (5.1)
Health score	87.7 (14.8)	90.8 (11.5)	90.2 (13.5)	85.9 (13.8)	86.0 (15.9)	88.2 (13.9)	88.6 (13.8)
**Female**							
Total, N (%)	15125 (18.2)	29464 (35.5)	3663 (4.4)	9847 (11.9)	12787 (15.4)	12057 (14.5)	82943 (100)
Human development index	0.47 (0.10)	0.63 (0.04)	0.48 (0.08)	0.71 (0.02)	0.43 (0.09)	0.55 (0.09)	0.56 (0.12)
Age, years	42.5 (14.6)	44.9 (15.2)	42.5 (14.2)	50 (15.7)	42.8 (13.8)	43.5 (13.7)	44.4 (14.9)
Education, years	5.7 (4.9)	6.6 (5.0)	3.5 (5.1)	12.0 (3.6)	4.1 (4.6)	7.0 (4.5)	6.6 (5.2)
Health score	83.8 (16.1)	87.5 (12.9)	83.5 (17.0)	81.3 (15.6)	82.0 (18.0)	86.8 (14.2)	84.9 (15.3)

Table S4 Effect estimate (95% Confidence Interval) for human development level (comparing 0.672 to 0.572) on individual health (sensitivity analysis), World Health Survey 2002-2004 (N=148679).

	Male	Female
Total effect	1.54 (-0.74, 3.83)	2.42 (0.44, 4.41)
Pure direct effect	1.29 (-0.99, 3.57)	2.00 (0.02, 3.98)
Total indirect effect	0.26 (0.17, 0.34)	0.42 (0.30, 0.55)

Table S5 Potential confounders for BMI-health relation[a] by WHO region, World Health Survey 2002-2004 (N=105630).

**Characteristics**, mean (SD)	**Africa**	**The Americas**	**Eastern Mediterranean**	**Europe**	**South-East Asia**	**Western Pacific**	**All**
Living in rural areas, N (%)	13240 (61.55)	11093 (31.66)	1815 (42.14)	3398 (27.38)	11313 (75.19)	10513 (53.22)	51372 (47.54)
Unemployment, N (%)	8710 (40.49)	15602 (44.52)	1993 (46.27)	4905 (39.52)	4951 (32.91)	6390 (32.35)	42551 (39.37)
Not Married, N (%)	7635 (35.5)	15485 (44.19)	866 (20.11)	4809 (38.75)	3037 (20.18)	3553 (17.99)	35385 (32.74)
Currently smoking, N (%)	3616 (16.81)	8031 (22.92)	1215 (28.21)	3396 (27.36)	5418 (36.01)	6619 (33.51)	28295 (26.18)
Alcohol use, N (%)	6926 (32.2)	19785 (56.46)	308 (7.15)	9700 (78.16)	2900 (19.27)	7433 (37.63)	47052 (43.54)
Physical inactivity, N (%)	13663 (63.52)	28328 (80.84)	3379 (78.45)	9061 (73.01)	8630 (57.36)	12858 (65.1)	75919 (70.25)

[a] Factors examined in sensitivity analyses and their statistics were from sample further restricted to individuals have complete information on these factors.

Supplementary Figures

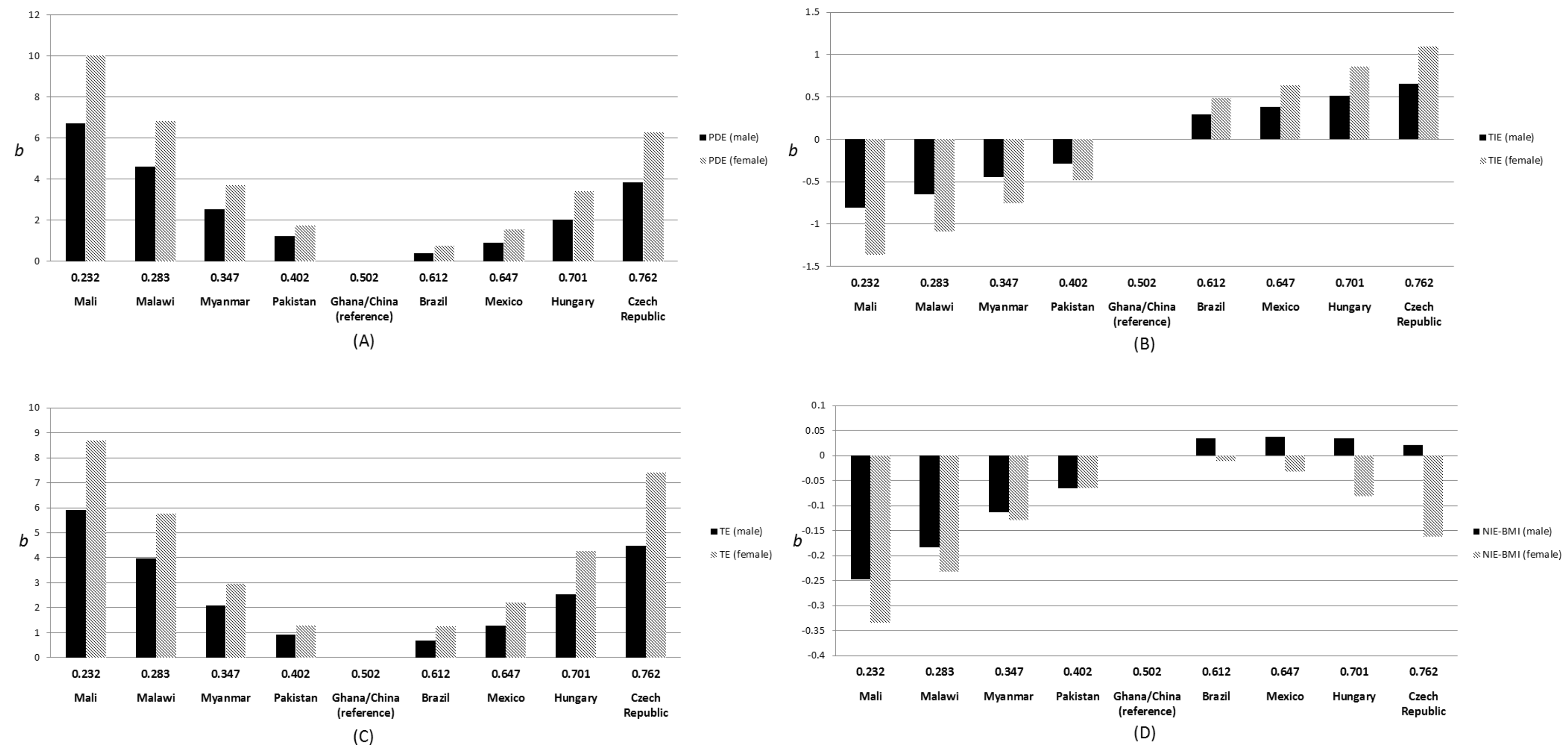

Figure S1. Sex specific effect estimates when comparing countries with different HDI levels to Ghana/China (reference, HDI=0.502), obtained from multilevel regression analysis of the World Health Survey 2002-2004 (N=109448). Pure direct effect (PDE; A), total indirect effect (TIE; B), and total effect (TE; C) of HDI on health were obtained when education was the mediator of interest in Scenario 1. Natural indirect effect via BMI only was obtained when BMI was the mediator of interest in Scenario 2 (D). Y axis represents mean difference in health score when comparing a certain country to Ghana/China.

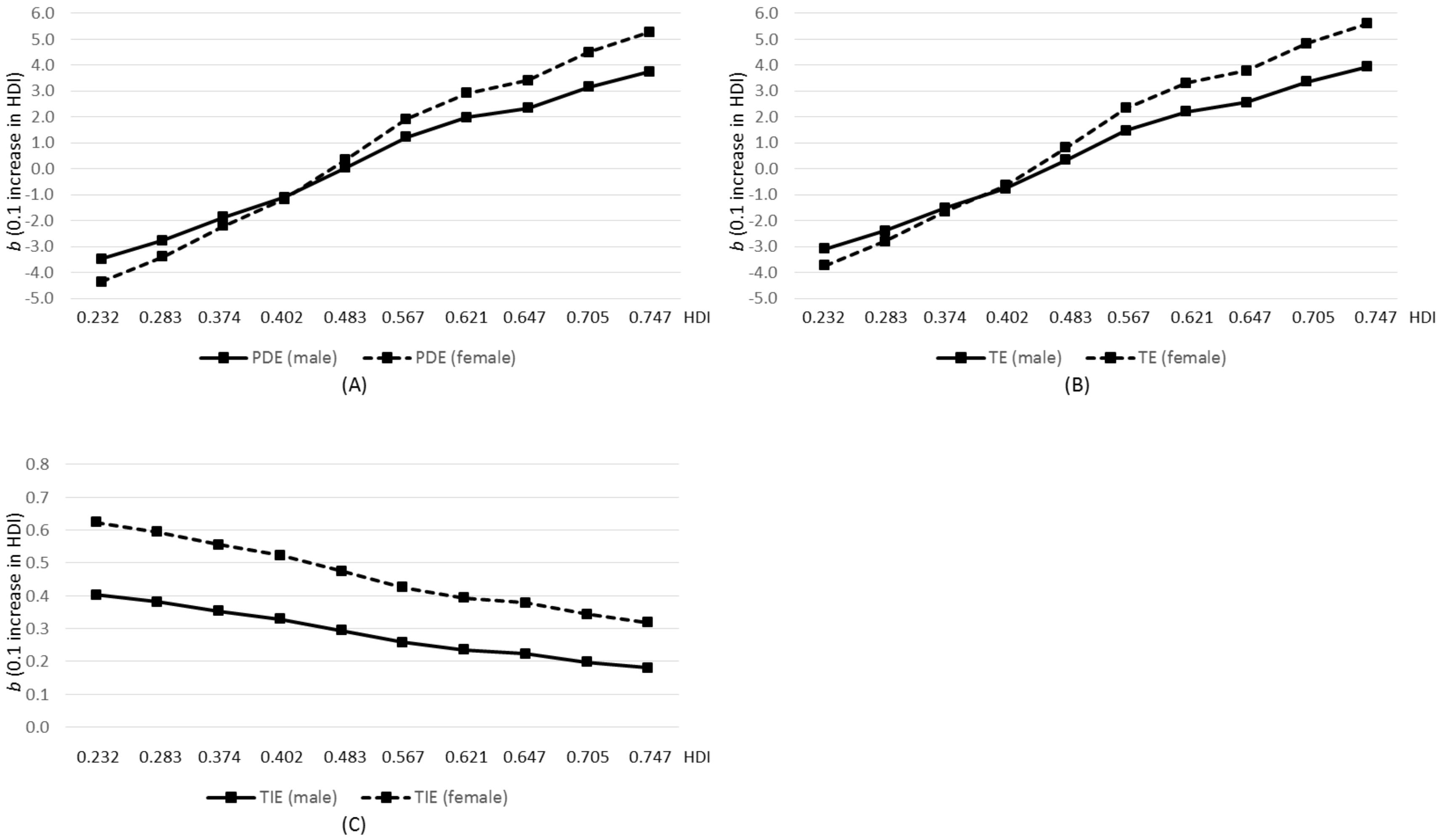

Figure S2. Pure direct effect (PDE; A), total indirect effect (TIE; B), and total effect (TE) of HDI on health when education is the mediator of interest in Scenario 1, obtained from multilevel regression analysis of the World Health Survey 2002-2004 (N=148679). Y axis represents mean difference in health score associated 0.1-unit increase in HDI. X axis represents selected HDI values within the range of the current sample.

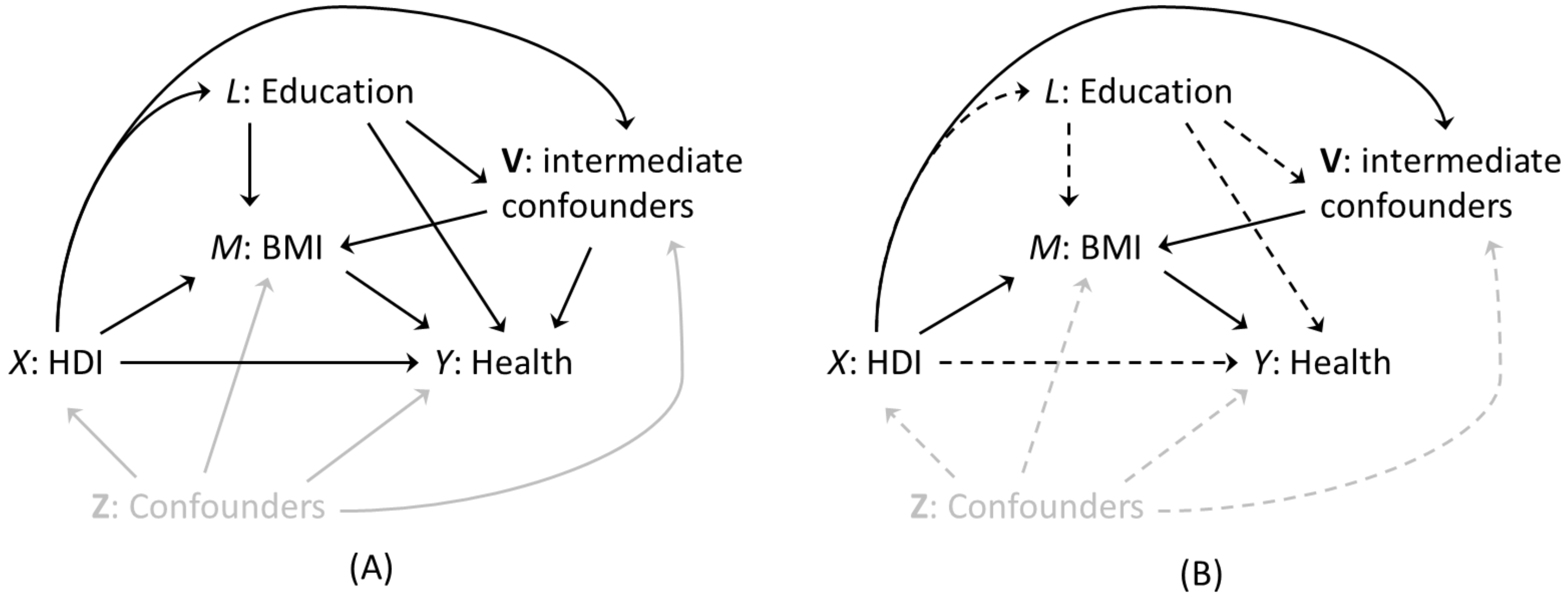

Figure S3. Graphical representation of scenario 2 in the presence of *M*-*Y* confounder set *V* that was affected by *X* and *L* (A). These confounders included living in urban areas, marital status, unemployment, smoking, alcohol use, and physical inactivity. In figure S2 (B), by implementing a g-estimation-like method to achieve conditional independence of *V* and *Y*, we can estimate the indirect effect of HDI on health via BMI but not education (solid line).

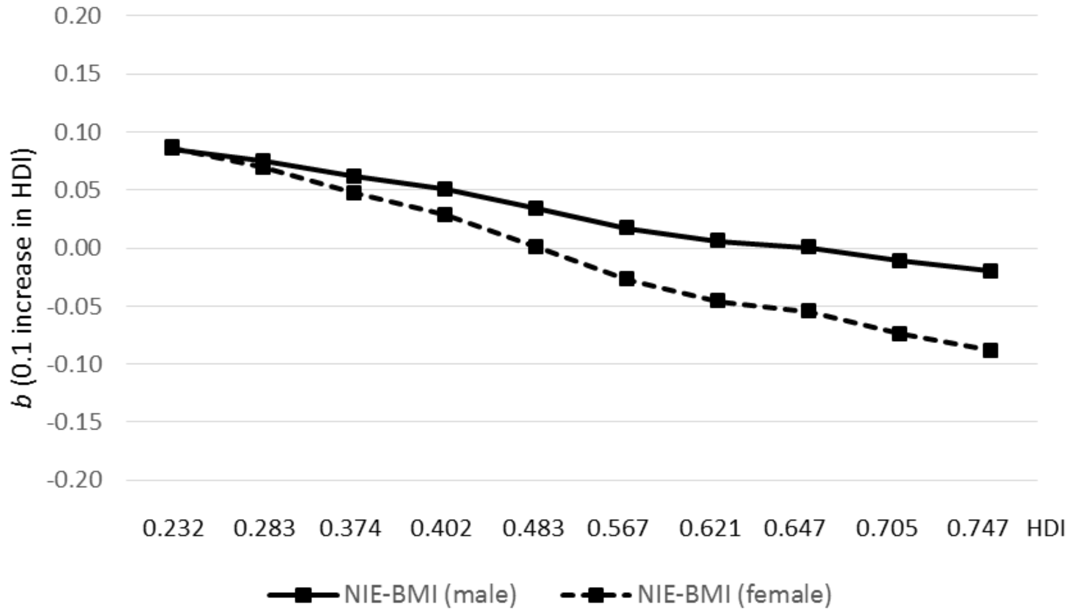

Figure S4. Results from sensitivity analyses for natural indirect effect via BMI only in Scenario 2, obtained from multilevel regression analysis of the World Health Survey 2002-2004 (N=109448). Y axis represents mean difference in health score associated with a 0.1-unit increase in HDI. X axis represents selected reference HDI values within the range of the current sample.

Reference

Daniel RM., De Stavola BL., Cousens SN., Vansteelandt S. 2015. Causal mediation analysis with multiple mediators. *Biometrics* 71:1–14. DOI: 10.1111/biom.12248.

Rubin DB. 1980. Discussion of "Randomization analysis of experimental data in the Fisher randomization test" by Basu. *Journal of the American Statistical Association* 75:591–593.

Rubin DB. 1990. Neyman (1923) and causal inference in experiments and observational studies. *Statistical Science* 5:472–480.
