# Supplementary material for: The impact of human development on individual health: a causal mediation analysis examining pathways through education and body mass index
Source: PeerJ. 2017 Mar 1;5:e3053. doi: 10.7717/peerj.3053 (PMC5335675; doi:10.7717/peerj.3053)
Supplement: Supplemental Information 2 [file peerj-05-3053-s002.pdf]

```
/*=======================================================================================
Manuscript title:
       The impact of human development on individual’s health: a causal
       mediation analysis examining pathways through education and body mass index

Program created by: Aolin Wang <aolinw@ucla.edu>

Data: WHS Individual dataset (ALL COUNTRIES) + HDI
Date Created: 9/24/2015



Main Analysis (N=109448):

1. Scenario1: HDI on health, education as the only mediator

Exposure: HDI
Mediator: education years
Outcome: health score (PCA)
X-M-Y confounder: whoregion (afr emr eur sear wpr)
M-Y confounder: age age2 fem

Purpose: choose 8 points for HDI as x0 and examine 1-unit increase -- plot
       X-axis: HDI
       Y-axis: effect
       Line: by sex

2. Scenario2: HDI on health, BMI as the mediator of interest

Exposure: HDI
X-induced M-Y confounder (L): education years
Mediator: BMI
Outcome: health score (PCA)
X-L-M-Y confounder: whoregion (afr emr eur sear wpr)
L-M-Y confounder: age age2 fem
M-Y confounder: urban, married (can be affected by L)

Note: the decomposition follow's VanderWeele's 2014 paper where the
       NIE-BMI is part of the PDE if BMI is ignored.
       NIE-edu will be the same as TIE(via edu).


Sensitivity Analysis:

1. For Scenario 1:
       Relaxed the sample restriction criteria to individuals with complete information
       on HDI, education, and health score (N=148679).
       Same programming code was used.

2. For Scenario 2:
       Further examined the robustness of our result in the presence of intermediate
       confounders V (affected by HDI, education or both) of the BMI-health relationship.
       (N=105630)

       Method: We used g-estimation technique to create a confounding-free outcome
       variable where this new outcome variable is independent of V conditional on HDI,
       education, BMI, and other covariates (the set Z).
       See Supplementary file for details.
 =======================================================================================*/

%let d= C:\Users\Aolin\Dropbox\HDI mediation\Data;
libname d "&d";
title;
options fmtsearch=(d) nofmterr formdlim='-' helpbrowser=sas mprint;
options pageno=1 nodate threads=yes cpucount=actual;
ods graphics off;
```

```
/**********************************************************
Main analysis:
restrict to sample with complete info on HDI, Edu, and cov
plus BMI (exclude extreme ht and wt and BMI<14)
**********************************************************/

/**********************************************************
 Scenario1: HDI on health, education as the only mediator
**********************************************************/

data ana1; set d.ana1(encoding='asciiany');
where normhtwt=1;
y=hlth;
x=hdi;
x2=hdi*hdi;
xm=hdi*eduyr5; *for ease of coding and change var later;
m=eduyr5;
run;
%let cov1 = age age2 fem afr emr eur sear wpr;
%let dsn = ana1;
proc sort data=&dsn; by whocode; run;

proc freq data=ana1;
table hdi hdi90un;
run;

/*
sex-specific
*/
%let cov1 = age age2 afr emr eur sear wpr;

*** Male;
/* Model for Y */
proc mixed data=&dsn method=REML noitprint;
where fem=0;
class whocode;
model y = x x2 m xm &cov1 /solution;
random intercept/sub=whocode type=un;
ods output solutionF=paraYm;
run;

/* Model for M */
proc mixed data=&dsn method=REML noitprint;
where fem=0;
class whocode;
model m = x &cov1 /solution;
random intercept/sub=whocode type=un;
ods output solutionF=paraMm;
run;

proc sql noprint;
select Estimate into : g1- :g12
from paraYm
where monotonic() between 1 and 12; quit;
proc sql noprint;
select Estimate into : b1- :b9
from paraMm
where monotonic() between 1 and 9; quit;

proc sql;
select mean(afr), mean(emr), mean(eur), mean(sear), mean(wpr)
       into :m_afr, :m_emr, :m_eur, :m_sear, :m_wpr
from &dsn
where fem=0; quit;
```

```
%macro eduyr_male (index=, ref=);
proc  nlmixed  data=&dsn;
where fem=0;
parms
gY=&g1 gX=&g2 gX2=&g3 gM=&g4 gXM=&g5
       gZ1=&g6 gZ2=&g7 gZ4=&g8 gZ5=&g9 gZ6=&g10 gZ7=&g11 gZ8=&g12
bM=&b1 bX=&b2
       bZ1=&b3 bZ2=&b4 bZ4=&b5 bZ5=&b6 bZ6=&b7 bZ7=&b8 bZ8=&b9;

x1=&index; x0=&ref; mstar=0; cZ1=0; /*age=45*/ cZ2=0; /*age square*/
cZ4=&m_afr; cZ5=&m_emr; cZ6=&m_eur; cZ7=&m_sear; cZ8=&m_wpr; /*AMR*/

mu_y = (gY + uY) + gX*X + gX2*X2 + gM*M + gXM*XM + gZ1*age + gZ2*age2
              + gZ4*afr + gZ5*emr + gZ6*eur + gZ7*sear + gZ8*wpr ;
mu_m = (bM + uM) + bX*X + bZ1*age + bZ2*age2
              + bZ4*afr + bZ5*emr + bZ6*eur + bZ7*sear + bZ8*wpr ;
loglik_y=  -0.5*((y-mu_y)**2)/(ss_y)-0.5*log(ss_y);
loglik_m=  -0.5*((m-mu_m)**2)/(ss_m)-0.5*log(ss_m);
ll_o=  loglik_m  +  loglik_y;
model  Y  ~ general(ll_o);
random uY uM ~ normal([0,0], [s2uY, 0, s2uM]) subject=whocode;

bcc  =  bZ1*cZ1 + bZ2*cZ2 + bZ4*cZ4 + bZ5*cZ5 + bZ6*cZ6
              + bZ7*cZ7 + bZ8*cZ8; /*bcc is a constant, not depend on covariates once set
at top*/
cde = (gX + gXM*mstar)*(x1-x0) + gX2*(x1*x1-x0*x0);
rie = gXM*(bM + bX*x0 + bcc - mstar)*(x1-x0);
mie = gXM*bX*(x1-x0)*(x1-x0);
pie = (gXM*bX*x0 + gM*bX)*(x1-x0);
tde = cde + rie + mie;
pde = cde + rie;
tie = pie + mie;
te  = cde + rie + mie + pie;
estimate      "TE(M)_&ref"  te;
estimate      "PDE(M)_&ref" cde+rie;
estimate      "TIE(M)_&ref" pie+mie;
estimate      "CDE(M)_&ref" cde;
estimate      "RIE(M)_&ref" rie;
estimate      "MIE(M)_&ref" mie;
estimate      "PIE(M)_&ref" pie;
estimate      "TDE(M)_&ref" cde+rie+mie;
run;
%mend eduyr_male;

%eduyr_male (index=0.83, ref=-0.17);

%eduyr_male (index=-2.57, ref=-3.57);
%eduyr_male (index=-2.06, ref=-3.06);
%eduyr_male (index=-1.42, ref=-2.42);
%eduyr_male (index=-0.87, ref=-1.87);
%eduyr_male (index=-0.06, ref=-1.06);
%eduyr_male (index=0.78, ref=-0.22);
%eduyr_male (index=1.32, ref=0.32);
%eduyr_male (index=1.58, ref=0.58);
%eduyr_male (index=2.16, ref=1.16);
%eduyr_male (index=2.58, ref=1.58);


*** Female;
/* Model for Y */
proc mixed data=&dsn method=REML noitprint;
where fem=1;
class whocode;
model y = x x2 m xm &cov1 /solution;
random intercept/sub=whocode type=un;
ods output solutionF=paraYf;
run;
```

```
/* Model for M */
proc mixed data=&dsn method=REML noitprint;
where fem=1;
class whocode;
model m = x &cov1 /solution;
random intercept/sub=whocode type=un;
ods output solutionF=paraMf;
run;

proc sql noprint;
select Estimate into : g1- :g12
from paraYf
where monotonic() between 1 and 12; quit;
proc sql noprint;
select Estimate into : b1- :b9
from paraMf
where monotonic() between 1 and 9; quit;

proc sql;
select mean(afr), mean(emr), mean(eur), mean(sear), mean(wpr)
       into :m_afr, :m_emr, :m_eur, :m_sear, :m_wpr
from &dsn
where fem=1; quit;

%macro eduyr_female (index=, ref=);
proc  nlmixed  data=&dsn;
where fem=1;
parms
gY=&g1 gX=&g2 gX2=&g3 gM=&g4 gXM=&g5
       gZ1=&g6 gZ2=&g7 gZ4=&g8 gZ5=&g9 gZ6=&g10 gZ7=&g11 gZ8=&g12
bM=&b1 bX=&b2
       bZ1=&b3 bZ2=&b4 bZ4=&b5 bZ5=&b6 bZ6=&b7 bZ7=&b8 bZ8=&b9;

x1=&index; x0=&ref; mstar=0; cZ1=0; /*age=45*/ cZ2=0; /*age square*/
cZ4=&m_afr; cZ5=&m_emr; cZ6=&m_eur; cZ7=&m_sear; cZ8=&m_wpr; /*AMR*/

mu_y = (gY + uY) + gX*X + gX2*X2 + gM*M + gXM*XM + gZ1*age + gZ2*age2
              + gZ4*afr + gZ5*emr + gZ6*eur + gZ7*sear + gZ8*wpr ;
mu_m = (bM + uM) + bX*X + bZ1*age + bZ2*age2
              + bZ4*afr + bZ5*emr + bZ6*eur + bZ7*sear + bZ8*wpr ;
loglik_y=  -0.5*((y-mu_y)**2)/(ss_y)-0.5*log(ss_y);
loglik_m=  -0.5*((m-mu_m)**2)/(ss_m)-0.5*log(ss_m);
ll_o=  loglik_m  +  loglik_y;
model  Y  ~ general(ll_o);
random uY uM ~ normal([0,0], [s2uY, 0, s2uM]) subject=whocode;

bcc  =  bZ1*cZ1 + bZ2*cZ2 + bZ4*cZ4 + bZ5*cZ5 + bZ6*cZ6
              + bZ7*cZ7 + bZ8*cZ8; /*bcc is a constant, not depend on covariates once set
at top*/
cde = (gX + gXM*mstar)*(x1-x0) + gX2*(x1*x1-x0*x0);
rie = gXM*(bM + bX*x0 + bcc - mstar)*(x1-x0);
mie = gXM*bX*(x1-x0)*(x1-x0);
pie = (gXM*bX*x0 + gM*bX)*(x1-x0);
tde = cde + rie + mie;
pde = cde + rie;
tie = pie + mie;
te  = cde + rie + mie + pie;
estimate      "TE(F)_&ref"  te;
estimate      "PDE(F)_&ref" cde+rie;
estimate      "TIE(F)_&ref" pie+mie;
estimate      "CDE(F)_&ref" cde;
estimate      "RIE(F)_&ref" rie;
estimate      "MIE(F)_&ref" mie;
estimate      "PIE(F)_&ref" pie;
estimate      "TDE(F)_&ref" cde+rie+mie;
run;
%mend eduyr_female;
```

```
%eduyr_female (index=0.83, ref=-0.17);

%eduyr_female (index=-2.57, ref=-3.57);
%eduyr_female (index=-2.06, ref=-3.06);
%eduyr_female (index=-1.42, ref=-2.42);
%eduyr_female (index=-0.87, ref=-1.87);
%eduyr_female (index=-0.06, ref=-1.06);
%eduyr_female (index=0.78, ref=-0.22);
%eduyr_female (index=1.32, ref=0.32);
%eduyr_female (index=1.58, ref=0.58);
%eduyr_female (index=2.16, ref=1.16);
%eduyr_female (index=2.58, ref=1.58);



/************************************************************
 Scenario2: HDI on health, BMI as the mediator of interest
************************************************************/

data ana1; set d.ana1(encoding='asciiany');
where normhtwt=1;
y=hlth;
x=hdi;
x2=hdi*hdi;
l=eduyr5;
m=bmic5;
xl=hdi*eduyr5; *for ease of coding and change var later;
xm=hdi*bmic5;
lm=eduyr5*bmic5;
mar = married;
smo = smoke;
run; *N=109448;

%let cov1 = age age2 afr emr eur sear wpr fem;
%let dsn = ana1;
proc sort data=&dsn; by whocode; run;

/*
sex-specific
*/
%let cov1 = age age2 afr emr eur sear wpr;

*** Male;
/* Model for Y when not accounting for urb, smo, and (mar, alc, pia) */
proc mixed data=&dsn method=REML noitprint;
where fem=0;
class whocode;
model y = x x2 l m xl xm lm &cov1 /solution;
random intercept/sub=whocode type=un;
ods output solutionF=paraY1m;
run;

/* Model for M */
proc mixed data=&dsn method=REML noitprint;
where fem=0;
class whocode;
model m = x l &cov1 /solution;
random intercept/sub=whocode type=un;
ods output solutionF=paraM1m;
run;

/* Model for L */
proc mixed data=&dsn method=REML noitprint;
where fem=0;
class whocode;
model l = x &cov1 /solution;
```

```
random intercept/sub=whocode type=un;
ods output solutionF=paraL1m;
run;


proc sql noprint;
select Estimate into : g1- :g15
from paraY1m
where monotonic() between 1 and 15; quit;
proc sql noprint;
select Estimate into : b1- :b10
from paraM1m
where monotonic() between 1 and 10; quit;
proc sql noprint;
select Estimate into : a1- :a9
from paraL1m
where monotonic() between 1 and 9; quit;

proc sql;
select  mean(afr), mean(emr), mean(eur), mean(sear), mean(wpr)
       into :m_afr, :m_emr, :m_eur, :m_sear, :m_wpr
from &dsn
where fem=0; quit;

%put _user_;

*male;
%macro bmi_male (index=, ref=);
proc  nlmixed  data=&dsn;
where fem=0;
parms
gY=&g1 gX=&g2 gX2=&g3 gL=&g4 gM=&g5 gXL=&g6 gXM=&g7 gLM=&g8
       gZ1=&g9 gZ2=&g10 gZ3=&g11 gZ4=&g12 gZ5=&g13 gZ6=&g14 gZ7=&g15
bM=&b1 bX=&b2 bL=&b3
       bZ1=&b4 bZ2=&b5 bZ3=&b6 bZ4=&b7 bZ5=&b8 bZ6=&b9 bZ7=&b10
aL=&a1 aX=&a2
       aZ1=&a3 aZ2=&a4 aZ3=&a5 aZ4=&a6 aZ5=&a7 aZ6=&a8 aZ7=&a9
;

x1=&index; x0=&ref; cZ1=0; /*age=45*/ cZ2=0; /*age2*/
cZ3=&m_afr; cZ4=&m_emr; cZ5=&m_eur; cZ6=&m_sear; cZ7=&m_wpr; /*mean WHOregion*/

mu_y = (gY + uY) + gX*X + gX2*X2 + gL*L + gM*M + gXL*XL + gXM*XM + gLM*LM
              + gZ1*age + gZ2*age2
              + gZ3*afr + gZ4*emr + gZ5*eur + gZ6*sear + gZ7*wpr ;
mu_m = (bM + uM) + bX*X + bL*L
              + bZ1*age + bZ2*age2
              + bZ3*afr + bZ4*emr + bZ5*eur + bZ6*sear + bZ7*wpr ;
mu_l = (aL + uL) + aX*X
              + aZ1*age + aZ2*age2
              + aZ3*afr + aZ4*emr + aZ5*eur + aZ6*sear + aZ7*wpr ;

loglik_y=  -0.5*((y-mu_y)**2)/(ss_y)-0.5*log(ss_y);
loglik_m=  -0.5*((m-mu_m)**2)/(ss_m)-0.5*log(ss_m);
loglik_l=  -0.5*((l-mu_l)**2)/(ss_l)-0.5*log(ss_l);

ll_o=  loglik_l + loglik_m  +  loglik_y;
model  Y  ~ general(ll_o);

random uY uM uL ~ normal([0,0,0], [s2uY, 0, s2uM, 0, 0, s2uL]) subject=whocode;

bcc = (bZ1*cZ1 + bZ2*cZ2 + bZ3*cZ3 + bZ4*cZ4 + bZ5*cZ5 + bZ6*cZ6
              + bZ7*cZ7); /*Z in eq for M*/
acc = (aZ1*cZ1 + aZ2*cZ2 + aZ3*cZ3 + aZ4*cZ4 + aZ5*cZ5 + aZ6*cZ6
              + aZ7*cZ7); /*Z in eq for L*/

NDE    = (    gX + gXL*aL + gXM*(bM + bL*aL)
```

```
              + x0*(gXL*aX + gXM*bL*aX)
              + x0*(gXM*bX)
              + acc*(gXL + gXM*bL)
              + bcc*gXM )*(x1-x0)
              + gX2*(x1*x1-x0*x0);
NIE1= (aX*(gL + gM*bL + gLM*(bM + 2*aL*bL))
              + x1*aX*(gXL + gXM*bL)
              + x1*aX*bX*gLM
              + acc*aX*gLM*bL
              + 2*bcc*aX*gLM )*(x1-x0)
              + aX*aX*bL*gLM*(x1*x1-x0*x0);
NIE2= (bX*(gM + gLM*aL)
              + x1*bX*gXM
              + x0*bX*aX*gLM
              + acc*bX*gLM )*(x1-x0);

te = nde + nie1 + nie2;
pde = nde + nie2;
estimate      "TE_&ref"      te;
estimate      "NDE_&ref"     nde;
estimate      "NIE(X-LY)_&ref"      nie1;
estimate      "NIE(X-M-Y)_&ref"     nie2;
estimate      "PDE_&ref"     pde;
run;
%mend bmi_male;

%bmi_male (index=0.83, ref=-0.17);

%bmi_male (index=-2.57, ref=-3.57);
%bmi_male (index=-2.06, ref=-3.06);
%bmi_male (index=-1.42, ref=-2.42);
%bmi_male (index=-0.87, ref=-1.87);
%bmi_male (index=-0.06, ref=-1.06);
%bmi_male (index=0.78, ref=-0.22);
%bmi_male (index=1.32, ref=0.32);
%bmi_male (index=1.58, ref=0.58);
%bmi_male (index=2.16, ref=1.16);
%bmi_male (index=2.58, ref=1.58);


*** Female;
/* Model for Y when not accounting for urb, smo, and (mar, alc, pia) */
proc mixed data=&dsn method=REML noitprint;
where fem=1;
class whocode;
model y = x x2 l m xl xm lm &cov1 /solution;
random intercept/sub=whocode type=un;
ods output solutionF=paraY1f;
run;

/* Model for M */
proc mixed data=&dsn method=REML noitprint;
where fem=1;
class whocode;
model m = x l &cov1 /solution;
random intercept/sub=whocode type=un;
ods output solutionF=paraM1f;
run;

/* Model for L */
proc mixed data=&dsn method=REML noitprint;
where fem=1;
class whocode;
model l = x &cov1 /solution;
random intercept/sub=whocode type=un;
ods output solutionF=paraL1f;
run;
```

```
proc sql noprint;
select Estimate into : g1- :g15
from paraY1f
where monotonic() between 1 and 15; quit;
proc sql noprint;
select Estimate into : b1- :b10
from paraM1f
where monotonic() between 1 and 10; quit;
proc sql noprint;
select Estimate into : a1- :a9
from paraL1f
where monotonic() between 1 and 9; quit;

proc sql;
select  mean(afr), mean(emr), mean(eur), mean(sear), mean(wpr)
       into :m_afr, :m_emr, :m_eur, :m_sear, :m_wpr
from &dsn
where fem=1; quit;

%put _user_;

*female;
%macro bmi_female (index=, ref=);
proc  nlmixed  data=&dsn;
where fem=1;
parms
gY=&g1 gX=&g2 gX2=&g3 gL=&g4 gM=&g5 gXL=&g6 gXM=&g7 gLM=&g8
       gZ1=&g9 gZ2=&g10 gZ3=&g11 gZ4=&g12 gZ5=&g13 gZ6=&g14 gZ7=&g15
bM=&b1 bX=&b2 bL=&b3
       bZ1=&b4 bZ2=&b5 bZ3=&b6 bZ4=&b7 bZ5=&b8 bZ6=&b9 bZ7=&b10
aL=&a1 aX=&a2
       aZ1=&a3 aZ2=&a4 aZ3=&a5 aZ4=&a6 aZ5=&a7 aZ6=&a8 aZ7=&a9
;

x1=&index; x0=&ref; cZ1=0; /*age=45*/ cZ2=0; /*age2*/
cZ3=&m_afr; cZ4=&m_emr; cZ5=&m_eur; cZ6=&m_sear; cZ7=&m_wpr; /*mean WHOregion*/

mu_y = (gY + uY) + gX*X + gX2*X2 + gL*L + gM*M + gXL*XL + gXM*XM + gLM*LM
              + gZ1*age + gZ2*age2
              + gZ3*afr + gZ4*emr + gZ5*eur + gZ6*sear + gZ7*wpr ;
mu_m = (bM + uM) + bX*X + bL*L
              + bZ1*age + bZ2*age2
              + bZ3*afr + bZ4*emr + bZ5*eur + bZ6*sear + bZ7*wpr ;
mu_l = (aL + uL) + aX*X
              + aZ1*age + aZ2*age2
              + aZ3*afr + aZ4*emr + aZ5*eur + aZ6*sear + aZ7*wpr ;

loglik_y=  -0.5*((y-mu_y)**2)/(ss_y)-0.5*log(ss_y);
loglik_m=  -0.5*((m-mu_m)**2)/(ss_m)-0.5*log(ss_m);
loglik_l=  -0.5*((l-mu_l)**2)/(ss_l)-0.5*log(ss_l);

ll_o=  loglik_l + loglik_m  +  loglik_y;
model  Y  ~ general(ll_o);

random uY uM uL ~ normal([0,0,0], [s2uY, 0, s2uM, 0, 0, s2uL]) subject=whocode;

bcc = (bZ1*cZ1 + bZ2*cZ2 + bZ3*cZ3 + bZ4*cZ4 + bZ5*cZ5 + bZ6*cZ6
              + bZ7*cZ7); /*Z in eq for M*/
acc = (aZ1*cZ1 + aZ2*cZ2 + aZ3*cZ3 + aZ4*cZ4 + aZ5*cZ5 + aZ6*cZ6
              + aZ7*cZ7); /*Z in eq for L*/

NDE    = (    gX + gXL*aL + gXM*(bM + bL*aL)
              + x0*(gXL*aX + gXM*bL*aX)
              + x0*(gXM*bX)
              + acc*(gXL + gXM*bL)
              + bcc*gXM )*(x1-x0)
```

```
              + gX2*(x1*x1-x0*x0);
NIE1= (aX*(gL + gM*bL + gLM*(bM + 2*aL*bL))
              + x1*aX*(gXL + gXM*bL)
              + x1*aX*bX*gLM
              + acc*aX*gLM*bL
              + 2*bcc*aX*gLM )*(x1-x0)
              + aX*aX*bL*gLM*(x1*x1-x0*x0);
NIE2= (bX*(gM + gLM*aL)
              + x1*bX*gXM
              + x0*bX*aX*gLM
              + acc*bX*gLM )*(x1-x0);

te = nde + nie1 + nie2;
pde = nde + nie2;
estimate      "TE_&ref"      te;
estimate      "NDE_&ref"     nde;
estimate      "NIE(X-LY)_&ref"      nie1;
estimate      "NIE(X-M-Y)_&ref"     nie2;
estimate      "PDE_&ref"     pde;
run;
%mend bmi_female;

%bmi_female (index=0.83, ref=-0.17);

%bmi_female (index=-2.57, ref=-3.57);
%bmi_female (index=-2.06, ref=-3.06);
%bmi_female (index=-1.42, ref=-2.42);
%bmi_female (index=-0.87, ref=-1.87);
%bmi_female (index=-0.06, ref=-1.06);
%bmi_female (index=0.78, ref=-0.22);
%bmi_female (index=1.32, ref=0.32);
%bmi_female (index=1.58, ref=0.58);
%bmi_female (index=2.16, ref=1.16);
%bmi_female (index=2.58, ref=1.58);



/**********************************************************************************
Sensitivity analysis:

Scenario 2:
       Method: We used g-estimation technique to create a confounding-free outcome
       variable where this new outcome variable is independent of V conditional on HDI,
       education, BMI, and other covariates (the set Z).

**********************************************************************************/

data ana1; set d.ana1(encoding='asciiany');
where normhtwt=1 and nmiss_poconf=0;
y=hlth;
x=hdi;
x2=hdi*hdi;
l=eduyr5;
m=bmic5;
xl=hdi*eduyr5;
xm=hdi*bmic5;
lm=eduyr5*bmic5;
mar = married;
smo = smoke;
nma = 1-married;
nem = 1-employ;
run; *N=105630;

%let cov1 = age age2 afr emr eur sear wpr fem;
%let cov2 = urb nem nma;
%let cov3 = smo alc pia;
%let dsn = ana2;
```

```
proc sort data=&dsn; by whocode; run;

proc mixed data=&dsn method=REML noitprint;
class whocode;
model y = x x2 l m xl xm lm &cov1 &cov2 &cov3 /solution;
random intercept/sub=whocode type=un;
ods output solutionF=paraY3;
run;


/***********************
 Create seq g-estimator
 ***********************/
proc sql;
select  mean(urb), mean(nem), mean(nma),
              mean(smo), mean(alc), mean(pia)
       into :m_urb, :m_nem, :m_nma, :m_smo, :m_alc, :m_pia
from &dsn;
quit;

proc sql noprint;
select Estimate into : g1- :g6
from paraY3
where monotonic() between 17 and 22; quit;
%put _user_;

data &dsn; set &dsn;
Ynew = Y-&g1*(urb-&m_urb)-&g2*(nem-&m_nem)-&g3*(nma-&m_nma)
              -&g4*(smo-&m_smo)-&g5*(alc-&m_alc)-&g6*(pia-&m_pia);
proc means;
var Y Ynew;
run;

proc mixed data=&dsn method=REML noitprint;
class whocode;
model ynew = x x2 l m xl xm lm &cov1 /solution;
random intercept/sub=whocode type=un;
ods output solutionF=paraYnew1;
run;
* same coeff as from previous model adjusting for all covariates,
 intercept did changed (but int for Y model wouldn't affect effect
 estimation;

proc mixed data=&dsn method=REML noitprint;
class whocode;
model ynew = x x2 l m xl xm lm &cov1 &cov2 &cov3 /solution;
random intercept/sub=whocode type=un;
ods output solutionF=paraYnew3;
run; * coeff for &cov2 and &cov3 should be approximately zero;
*** checked, ok;


/*
sex-specific
*/
%let cov1 = age age2 afr emr eur sear wpr;
/*ods listing;*/
*** Male;
/* Model for Y, accounting for urb, smo, and (mar, alc, pia) */
proc mixed data=&dsn method=REML noitprint;
where fem=0;
class whocode;
model Ynew = x x2 l m xl xm lm &cov1 /solution;
random intercept/sub=whocode type=un;
ods output solutionF=paraY1m;
run;
```

```
/* Model for M */
proc mixed data=&dsn method=REML noitprint;
where fem=0;
class whocode;
model m = x l &cov1 /solution;
random intercept/sub=whocode type=un;
ods output solutionF=paraM1m;
run;

/* Model for L */
proc mixed data=&dsn method=REML noitprint;
where fem=0;
class whocode;
model l = x &cov1 /solution;
random intercept/sub=whocode type=un;
ods output solutionF=paraL1m;
run;


proc sql noprint;
select Estimate into : g1- :g15
from paraY1m
where monotonic() between 1 and 15; quit;
proc sql noprint;
select Estimate into : b1- :b10
from paraM1m
where monotonic() between 1 and 10; quit;
proc sql noprint;
select Estimate into : a1- :a9
from paraL1m
where monotonic() between 1 and 9; quit;

proc sql;
select  mean(afr), mean(emr), mean(eur), mean(sear), mean(wpr)
       into :m_afr, :m_emr, :m_eur, :m_sear, :m_wpr
from &dsn
where fem=0; quit;

%put _user_;

*male;
%macro bmi_male (index=, ref=);
proc  nlmixed  data=&dsn;
where fem=0;
parms
gY=&g1 gX=&g2 gX2=&g3 gL=&g4 gM=&g5 gXL=&g6 gXM=&g7 gLM=&g8
       gZ1=&g9 gZ2=&g10 gZ3=&g11 gZ4=&g12 gZ5=&g13 gZ6=&g14 gZ7=&g15
bM=&b1 bX=&b2 bL=&b3
       bZ1=&b4 bZ2=&b5 bZ3=&b6 bZ4=&b7 bZ5=&b8 bZ6=&b9 bZ7=&b10
aL=&a1 aX=&a2
       aZ1=&a3 aZ2=&a4 aZ3=&a5 aZ4=&a6 aZ5=&a7 aZ6=&a8 aZ7=&a9
;

x1=&index; x0=&ref; cZ1=0; /*age=45*/ cZ2=0; /*age2*/
cZ3=&m_afr; cZ4=&m_emr; cZ5=&m_eur; cZ6=&m_sear; cZ7=&m_wpr; /*mean WHOregion*/

mu_y = (gY + uY) + gX*X + gX2*X2 + gL*L + gM*M + gXL*XL + gXM*XM + gLM*LM
              + gZ1*age + gZ2*age2
              + gZ3*afr + gZ4*emr + gZ5*eur + gZ6*sear + gZ7*wpr ;
mu_m = (bM + uM) + bX*X + bL*L
              + bZ1*age + bZ2*age2
              + bZ3*afr + bZ4*emr + bZ5*eur + bZ6*sear + bZ7*wpr ;
mu_l = (aL + uL) + aX*X
              + aZ1*age + aZ2*age2
              + aZ3*afr + aZ4*emr + aZ5*eur + aZ6*sear + aZ7*wpr ;

loglik_y=  -0.5*((y-mu_y)**2)/(ss_y)-0.5*log(ss_y);
```

```
loglik_m=  -0.5*((m-mu_m)**2)/(ss_m)-0.5*log(ss_m);
loglik_l=  -0.5*((l-mu_l)**2)/(ss_l)-0.5*log(ss_l);

ll_o=  loglik_l + loglik_m  +  loglik_y;
model  Ynew  ~ general(ll_o);

random uY uM uL ~ normal([0,0,0], [s2uY, 0, s2uM, 0, 0, s2uL]) subject=whocode;

bcc = (bZ1*cZ1 + bZ2*cZ2 + bZ3*cZ3 + bZ4*cZ4 + bZ5*cZ5 + bZ6*cZ6
              + bZ7*cZ7); /*Z in eq for M*/
acc = (aZ1*cZ1 + aZ2*cZ2 + aZ3*cZ3 + aZ4*cZ4 + aZ5*cZ5 + aZ6*cZ6
              + aZ7*cZ7); /*Z in eq for L*/

NDE    = (    gX + gXL*aL + gXM*(bM + bL*aL)
              + x0*(gXL*aX + gXM*bL*aX)
              + x0*(gXM*bX)
              + acc*(gXL + gXM*bL)
              + bcc*gXM )*(x1-x0)
              + gX2*(x1*x1-x0*x0);
NIE1= (aX*(gL + gM*bL + gLM*(bM + 2*aL*bL))
              + x1*aX*(gXL + gXM*bL)
              + x1*aX*bX*gLM
              + acc*aX*gLM*bL
              + 2*bcc*aX*gLM )*(x1-x0)
              + aX*aX*bL*gLM*(x1*x1-x0*x0);
NIE2= (bX*(gM + gLM*aL)
              + x1*bX*gXM
              + x0*bX*aX*gLM
              + acc*bX*gLM )*(x1-x0);

te = nde + nie1 + nie2;
pde = nde + nie2;
estimate      "TE_&ref"      te;
estimate      "NDE_&ref"     nde;
estimate      "NIE(X-LY)_&ref"      nie1;
estimate      "NIE(X-M-Y)_&ref"     nie2;
estimate      "PDE_&ref"     pde;
run;
%mend bmi_male;

%bmi_male (index=0.83, ref=-0.17);

%bmi_male (index=-2.57, ref=-3.57);
%bmi_male (index=-2.06, ref=-3.06);
%bmi_male (index=-1.42, ref=-2.42);
%bmi_male (index=-0.87, ref=-1.87);
%bmi_male (index=-0.06, ref=-1.06);
%bmi_male (index=0.78, ref=-0.22);
%bmi_male (index=1.32, ref=0.32);
%bmi_male (index=1.58, ref=0.58);
%bmi_male (index=2.16, ref=1.16);
%bmi_male (index=2.58, ref=1.58);


*** Female;
/* Model for Y, accounting for urb, smo, and (mar, alc, pia) */
proc mixed data=&dsn method=REML noitprint;
where fem=1;
class whocode;
model Ynew = x x2 l m xl xm lm &cov1 /solution;
random intercept/sub=whocode type=un;
ods output solutionF=paraY1f;
run;

/* Model for M */
proc mixed data=&dsn method=REML noitprint;
where fem=1;
```

```
class whocode;
model m = x l &cov1 /solution;
random intercept/sub=whocode type=un;
ods output solutionF=paraM1f;
run;

/* Model for L */
proc mixed data=&dsn method=REML noitprint;
where fem=1;
class whocode;
model l = x &cov1 /solution;
random intercept/sub=whocode type=un;
ods output solutionF=paraL1f;
run;

proc sql noprint;
select Estimate into : g1- :g15
from paraY1f
where monotonic() between 1 and 15; quit;
proc sql noprint;
select Estimate into : b1- :b10
from paraM1f
where monotonic() between 1 and 10; quit;
proc sql noprint;
select Estimate into : a1- :a9
from paraL1f
where monotonic() between 1 and 9; quit;

proc sql;
select  mean(afr), mean(emr), mean(eur), mean(sear), mean(wpr)
       into :m_afr, :m_emr, :m_eur, :m_sear, :m_wpr
from &dsn
where fem=1; quit;

%put _user_;

*female;
%macro bmi_female (index=, ref=);
proc  nlmixed  data=&dsn;
where fem=1;
parms
gY=&g1 gX=&g2 gX2=&g3 gL=&g4 gM=&g5 gXL=&g6 gXM=&g7 gLM=&g8
       gZ1=&g9 gZ2=&g10 gZ3=&g11 gZ4=&g12 gZ5=&g13 gZ6=&g14 gZ7=&g15
bM=&b1 bX=&b2 bL=&b3
       bZ1=&b4 bZ2=&b5 bZ3=&b6 bZ4=&b7 bZ5=&b8 bZ6=&b9 bZ7=&b10
aL=&a1 aX=&a2
       aZ1=&a3 aZ2=&a4 aZ3=&a5 aZ4=&a6 aZ5=&a7 aZ6=&a8 aZ7=&a9
;

x1=&index; x0=&ref; cZ1=0; /*age=45*/ cZ2=0; /*age2*/
cZ3=&m_afr; cZ4=&m_emr; cZ5=&m_eur; cZ6=&m_sear; cZ7=&m_wpr; /*mean WHOregion*/

mu_y = (gY + uY) + gX*X + gX2*X2 + gL*L + gM*M + gXL*XL + gXM*XM + gLM*LM
              + gZ1*age + gZ2*age2
              + gZ3*afr + gZ4*emr + gZ5*eur + gZ6*sear + gZ7*wpr ;
mu_m = (bM + uM) + bX*X + bL*L
              + bZ1*age + bZ2*age2
              + bZ3*afr + bZ4*emr + bZ5*eur + bZ6*sear + bZ7*wpr ;
mu_l = (aL + uL) + aX*X
              + aZ1*age + aZ2*age2
              + aZ3*afr + aZ4*emr + aZ5*eur + aZ6*sear + aZ7*wpr ;

loglik_y=  -0.5*((y-mu_y)**2)/(ss_y)-0.5*log(ss_y);
loglik_m=  -0.5*((m-mu_m)**2)/(ss_m)-0.5*log(ss_m);
loglik_l=  -0.5*((l-mu_l)**2)/(ss_l)-0.5*log(ss_l);

ll_o=  loglik_l + loglik_m  +  loglik_y;
```

```
model  Ynew  ~ general(ll_o);

random uY uM uL ~ normal([0,0,0], [s2uY, 0, s2uM, 0, 0, s2uL]) subject=whocode;

bcc = (bZ1*cZ1 + bZ2*cZ2 + bZ3*cZ3 + bZ4*cZ4 + bZ5*cZ5 + bZ6*cZ6
              + bZ7*cZ7); /*Z in eq for M*/
acc = (aZ1*cZ1 + aZ2*cZ2 + aZ3*cZ3 + aZ4*cZ4 + aZ5*cZ5 + aZ6*cZ6
              + aZ7*cZ7); /*Z in eq for L*/

NDE    = (    gX + gXL*aL + gXM*(bM + bL*aL)
              + x0*(gXL*aX + gXM*bL*aX)
              + x0*(gXM*bX)
              + acc*(gXL + gXM*bL)
              + bcc*gXM )*(x1-x0)
              + gX2*(x1*x1-x0*x0);
NIE1= (aX*(gL + gM*bL + gLM*(bM + 2*aL*bL))
              + x1*aX*(gXL + gXM*bL)
              + x1*aX*bX*gLM
              + acc*aX*gLM*bL
              + 2*bcc*aX*gLM )*(x1-x0)
              + aX*aX*bL*gLM*(x1*x1-x0*x0);
NIE2= (bX*(gM + gLM*aL)
              + x1*bX*gXM
              + x0*bX*aX*gLM
              + acc*bX*gLM )*(x1-x0);

te = nde + nie1 + nie2;
pde = nde + nie2;
estimate      "TE_&ref"      te;
estimate      "NDE_&ref"     nde;
estimate      "NIE(X-LY)_&ref"      nie1;
estimate      "NIE(X-M-Y)_&ref"     nie2;
estimate      "PDE_&ref"     pde;
run;
%mend bmi_female;

%bmi_female (index=0.83, ref=-0.17);

%bmi_female (index=-2.57, ref=-3.57);
%bmi_female (index=-2.06, ref=-3.06);
%bmi_female (index=-1.42, ref=-2.42);
%bmi_female (index=-0.87, ref=-1.87);
%bmi_female (index=-0.06, ref=-1.06);
%bmi_female (index=0.78, ref=-0.22);
%bmi_female (index=1.32, ref=0.32);
%bmi_female (index=1.58, ref=0.58);
%bmi_female (index=2.16, ref=1.16);
%bmi_female (index=2.58, ref=1.58);
```
